# Observation of Multiple Ordered Solvation Shells in Doped Helium Droplets: The Case of He_*N*_Ca^2+^

**DOI:** 10.1021/acs.jpclett.3c00224

**Published:** 2023-03-23

**Authors:** Eva Zunzunegui-Bru, Elisabeth Gruber, Teresa Lázaro, Massimiliano Bartolomei, Marta I. Hernández, José Campos-Martínez, Tomás González-Lezana, Stefan Bergmeister, Fabio Zappa, Paul Scheier, Ricardo Pérez de Tudela, Javier Hernández-Rojas, José Bretón

**Affiliations:** † Instituto de Física Fundamental, IFF-CSIC, Serrano 123, Madrid 28006, Spain; ‡ Institut für Ionenphysik und Angewandte Physik, Universität Innsbruck, Technikerstraße 25, Innsbruck 6020, Austria; ¶ Independent Scientist, Wiemelhauser Straße 217, Bochum 44799, Germany; § Departamento de Física and IUdEA, Universidad de La Laguna, La Laguna, 38205, Tenerife, Spain

## Abstract

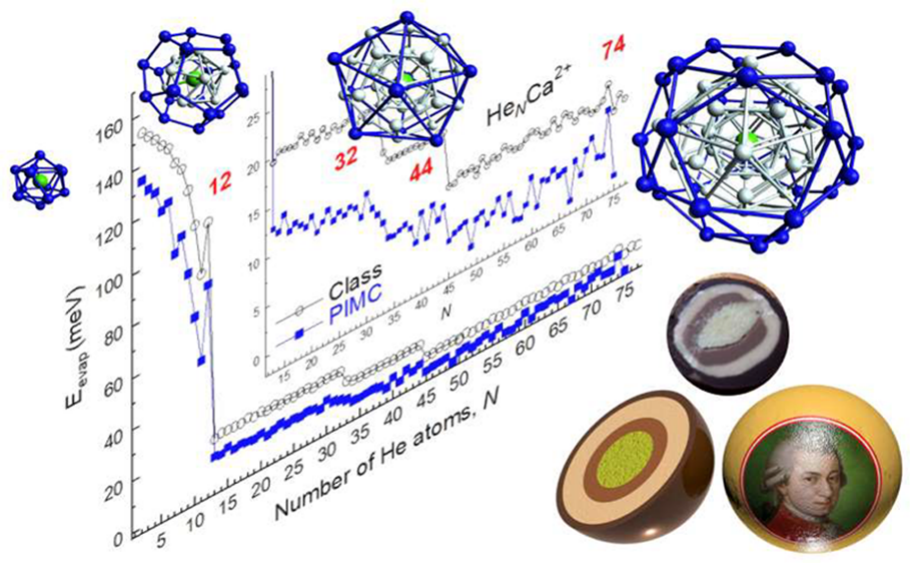

In this Letter, we report the experimental detection of likely the largest ordered structure of helium atoms surrounding a monatomic impurity observed to date using a recently developed technique. The mass spectrometry investigation of He_*N*_Ca^2+^ clusters, formed in multiply charged helium nanodroplets, reveals magic numbers at *N* = 12, 32, 44, and 74. Classical optimization and path integral Monte Carlo calculations suggest the existence of up to four shells surrounding the calcium dication which are closed with well-ordered Mozartkugel-like structures: He_12_Ca^2+^ with an icosahedron, the second at He_32_Ca^2+^ with a dodecahedron, the third at He_44_Ca^2+^ with a larger icosahedron, and finally for He_74_Ca^2+^, we find that the outermost He atoms form an icosidodecahedron which contains the other inner shells. We analyze the reasons for the formation of such ordered shells in order to guide the selection of possible candidates to exhibit a similar behavior.

The formation of clusters He_*N*_X where He atoms surround a central ion X forming discrete shells was proposed long time ago.^1,2^ Experiments using helium nanodroplets (HND),^3−11^ assisted by theoretical work,^12−20^ have served to find out which ions lead to the formation of solid-like snowballs^2^ or to liquid-like clusters. The abundance distributions of the different cluster sizes as a function of the number of He atoms, *N*, usually exhibit local anomalies (magic numbers) that correspond to particularly stable arrangements of the surrounding helium. The comparison of such observations with calculations of the evaporation energy (the energy required for the loss of a helium atom) and cluster structures is an effective manner to investigate if the formation of shells is the origin of such magic numbers, and in fact, this has been the procedure followed in some of our previous works on several doped HNDs.^20−22^

Among the various anomalies observed experimentally in the size dependence of abundances measured for a series of ions,^5,9,23^ the same sequence of magic numbers at *N* = 12, 32, and 44 has been detected for helium nanodroplets doped with Ar^+^,^9^ Ag^+^,^5^ and H_2_O^+^,^23^ and for molecular hydrogen droplets doped with Cs^+^^24^ and H^–^.^25^ Theoretical calculations showed that the abrupt features seen at those specific sizes for He_*N*_Ar^+^^11,19^ and (H_2_)_*N*_H^–^^26^ correlate with the closure of three consecutive solvation shells of icosahedral symmetry. These cases constitute, up to our knowledge, the largest number of such structures experimentally observed for HNDs to date.

In this work, we report the formation of four geometric solvation helium shells for He_*N*_Ca^2+^ obtained by means of a recently reported procedure to produce and study stable multiply charged HNDs,^27,28^ specially suitable for the detection of such multiple solid-like well ordered shells. As opposed to previous experiments, where helium droplets are first doped with neutral impurities and then ionized,^4,9,29^ HNDs, once ionized, are mass-to-charge selected prior to pickup of the neutral Ca, which eventually becomes doubly ionized inside the droplet. This new setup enables us to tune the specific size distribution of the droplets in a very efficient way. Alkali earth dopants constitute, in principle, ideal candidates to observe multiple solvation regions^5,30^ since their low ionization energies can lead to stable closed shell dications. Such species can strongly interact with He atoms due to an optimum balance between long-range induction and short-range exchange–repulsion interaction contributions. For Ca^2+^, classical optimization and quantum simulations help us to understand the growth pattern of the He_*N*_Ca^2+^ clusters, and we find that the sequence of ordered concentric shells are due not only to the stronger binding with the doubly ionized dopant but also to a “delicate” balance between the equilibrium distances of the He–He and He–dopant interacting couples.

The measurements were performed by using a recently developed setup^28^ (see Figure 1), which enables the observation of multiple solvation shells. Under the present conditions of the HND source (2.7 MPa, 9.7 K, resulting in droplets containing at average ∼10^6^ He atoms^3^), the electron ionizer (180 eV, 300 μA) and the settings of the sector field voltage, HNDs containing on average 10^5^ He atoms per charge with an average charge state of 5.3 are selected. In multiply charged HNDs, the charge centers are homogeneously distributed close to the surface of the droplets.^31^ Ca atoms that are picked up from the vapor produced in an ohmically heated oven (469 K) will be attracted by the charges and upon charge transfer can become singly and doubly charged (the sum of the first and second ionization energy of Ca is 17.98 eV which is below the potential energy of a charge center that can be considered to be a linear He_3_^+^, solvated by some He atoms^32^). The total yield of all He_*N*_Ca^2+^ is almost four times higher than the total yield of He_*N*_Ca^+^. This indicates that charge transfer from a He_*N*_^+^ charge center to Ca preferentially forms dications. The attachment of a second Ca atom to Ca^2+^ forms two singly charged Ca ions and Coulomb repulsion is expected to lead to the ejection of some of these swift ions from the HNDs. In contrast, the collision of neutral Ca atoms with Ca^+^ results in the formation of Ca_*n*_^+^ cluster ions. In order to maximize the yield of Ca^2+^, the vapor pressure of Ca in the pickup cell was set to a low value, i.e., the charge centers contain at average rather less than one Ca atom since we also observe many pristine He cluster ions.

**Figure 1:**
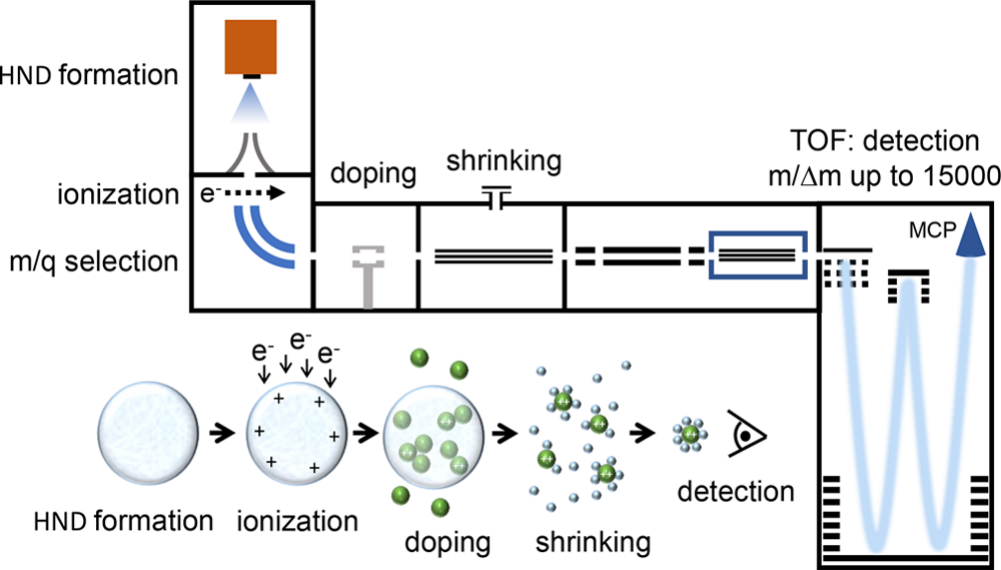
Schematic of the experimental setup. HNDs are multiply charged by electron impact and doped with Ca, evaporated in the oven in the pickup chamber. The charges across the HNDs act as nucleation sites and singly as well as doubly charged Ca ions are formed. In the evaporation chamber, equipped with an RF-hexapole and a gas inlet system, collisions between He gas at room temperature and the doped HNDs lead to the extraction of the trapped dopant ions, which are guided by further RF-multipole guides into the reflectron TOF-MS.

The newly formed Ca^+^ and Ca^2+^ are quickly solvated by neighboring He atoms that are bound by charge-induced dipole interactions. As described in ref (28), ions are gently extracted from multiply charged HNDs by shrinking the droplets in an evaporation chamber equipped with a radio frequency hexapole, via multiple collisions with room temperature He gas (at pressures *P*_evap_ = 0.07–0.13 Pa). Whenever the size of multiply charged HNDs shrinks below the critical size for a given charge state, a charge center solvated by He atoms is ejected from the droplets. Collisions of He gas with these ejected ions will reduce the number of He atoms solvating the ion. In this process weakly bound He atoms are first removed, which leads to intensity anomalies at numbers of He atoms *N* attached that have different evaporation energies compared to neighboring numbers. Shell closures will lead to pronounced intensity drops for larger sizes and magic numbers to exceptionally intense peaks at a given *N*. The appearance of especially stable structures is thereby not dependent on the evaporation pressure,^33^ however, when its value increases the maximum yield of He solvated ions shifts to lower He numbers which is essential for the identification of intensity anomalies at small values of *N*. Martini et al. recently demonstrated that He tagged dopant ions can also be formed efficiently upon surface collisions of charged and doped He droplets;^34^ however, this method does not allow the tuning of the average number of He atoms attached.

Eventually, the ions are registered by a time-of-flight mass-spectrometer, operated for the presented measurements in W-mode, enabling a high mass resolution of *m*/Δ*m* = 15000. The high mass resolution is necessary to clearly distinguish between He tagged dications He_*N*_Ca^2+^, He tagged singly charged ions He_*N*_Ca^+^, and pristine He_*N*_^+^ clusters. A section of a typical mass spectrum is plotted in Figure 2. Beside pristine He clusters, He tagged dications He_*N*_Ca^2+^ dominate under these conditions the spectrum. The peaks at odd masses arise from the pickup of water from the residual gas and 1 ppm impurity in the He gas used for shrinking the droplets. Since the binding energy of dications in HNDs is higher than that of singly charged ions, dications are expected to be solvated with more He atoms than monocations when they are ejected from HNDs. This agrees very well with the results where Ca^2+^ is solvated at average with 113 and Ca^+^ with 30 He atoms (for details see Figure S1 in the Supporting Information).

**Figure 2:**
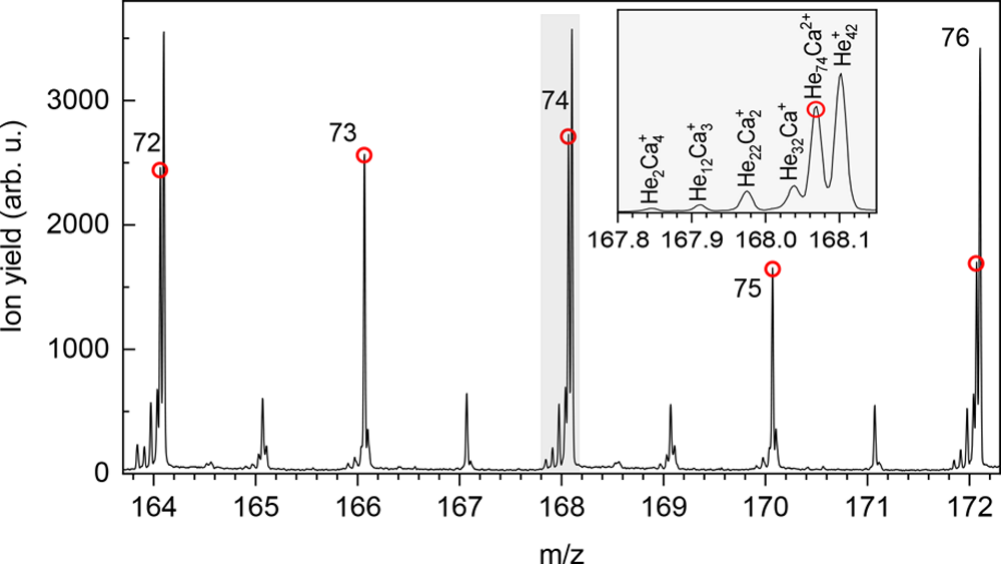
Section of a measured mass spectrum at *P*_evap_ of 0.11 Pa. The He tagged dications He_*N*_Ca^2+^ (highlighted by red circles for *N* = 72–76) are the dominant ion series beside the pristine He clusters. The peaks at odd masses arise from contaminations with water, here He_*N*_H_2_OCa^2+^ with *N* = 68–71. The high mass resolution of 15000 enables a straightforward evaluation of the mass spectrum, free from potential systematic errors due to impurities or isobaric ions. The zoom-in of the gray-marked peaks in the mass spectrum demonstrates the high quality of the experimental data.

The ion abundances of He_*N*_Ca^2+^ shown in Figure 3 were obtained from a mass spectrum with *P*_evap_ = 0.11 Pa and utilizing the custom designed software IsotopeFit,^35^ which takes the isotope pattern of the contributing ions into account. In contrast to previous studies where ionization of doped neutral HNDs was typically leading to monotonically decreasing ion abundances of He tagged ions as a function of *N*,^20,22^ the maximum of the size distribution of He_*N*_Ca^2+^ can be shifted to a desired value by setting the evaporation pressure *P*_evap_ to an appropiate value. Similar profiles in the corresponding ion abundances have been reported in recent studies of the He_*N*_SF_5_^+^ and He_*N*_SF_6_^+^ clusters.^33^ Peaks at *N* = 12, 32, 44, and 74 separate specific ranges of sizes (denoted as A, B, and C in Figure 3).

**Figure 3:**
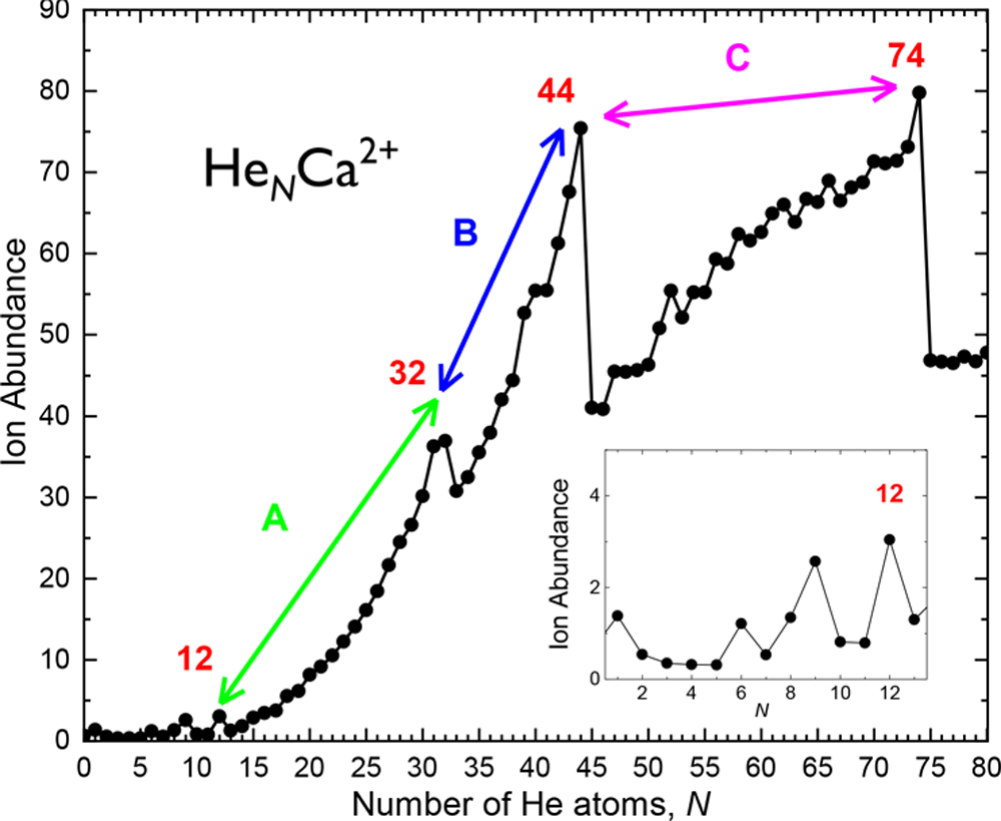
Experimental ion abundance for He_*N*_Ca^2+^ clusters as a function of the number of He atoms extracted from a mass spectrum taken at an evaporation pressure *P*_evap_ = 0.11 Pa of Figure 2. Different regions are identified between special features observed at *N* = 12, 32, 44, and 74. Inset is an amplified display of the region up to *N* = 13 He atoms. The higher ion abundance at *N* = 9 arises from contaminations with water, as the mass of He_9_Ca^2+^ is indistinguishable from that of (H_2_O)_2_Ca^2+^.

In order to properly predict the structural and energetic features of the He_*N*_Ca^2+^ clusters, we have built an accurate potential energy surface (PES) based on the sum of two-body (2B) and three-body (3B) noncovalent interaction contributions. The 2B contribution for the He–He interaction is adopted from ref (36), whereas for the He–Ca^2+^ interaction we have developed a new potential based on coupled cluster with single, double and perturbative triple excitation [CCSD(T)] results obtained by using the Molpro2012.1 package.^37^ In particular, accurate counterpoise corrected He–Ca^2+^ interaction energies have been computed by using the d-aug-cc-pV6Z^38^ and def2-AQZVPP^39^ basis sets for He and Ca^2+^, respectively. We have verified that the used basis set is large enough to guarantee well converged interaction energies, which are found to differ by less than 1% from those obtained in the minimum region with the d-aug-cc-pV5Z/def2-AQZVPP set. The He–Ca^2+^ 2B interaction is then analytically represented by means of an improved Lennard-Jones (ILJ) formulation^40^ and a 3B noncovalent contribution based on the dominant induced dipole–induced dipole interaction term.^20,41,42^ Further details and the corresponding analytical expressions of the PES employed in our calculations are shown in the Supporting Information.

With this PES, a combination of runs of classical optimization techniques such as the evolutionary algorithm (EA)^43^ and basin hopping (BH)^44^ methods have been carried out to obtain the minimum energy configurations for sizes of up to *N* = 78 He atoms. Values of the corresponding clusters energies per atom, *E*_*N*_/*N*, are shown in Figure 4. The trend followed by the *E*_*N*_/*N* curve indicates how the absolute value of the energy contained by each He atom within the cluster ion decreases as its size increases. Quantum mechanical (QM) calculations performed with both diffusion Monte Carlo (DMC)^45^ and Path Integral Monte Carlo (PIMC)^46^ at *T* = 2 K are also added in Figure 4. The qualitative agreement between the classical predictions and the QM values remains for the entire range of values *N* considered in this study. For *N* = 12, one of the prominent peaks observed in the experimental ion abundances (see Figure 3) in the low size droplets region, the energy per He atom curve, exhibits a noticeable feature. The classical optimization reveals that, for He_12_Ca^2+^, the minimum energy structure corresponds to an icosahedron formed with the He atoms surrounding the ionic impurity in the center (see inset in Figure 5). This was also the result found by Tramonto et al.^19^ for He_*N*_Ar^+^.

**Figure 4:**
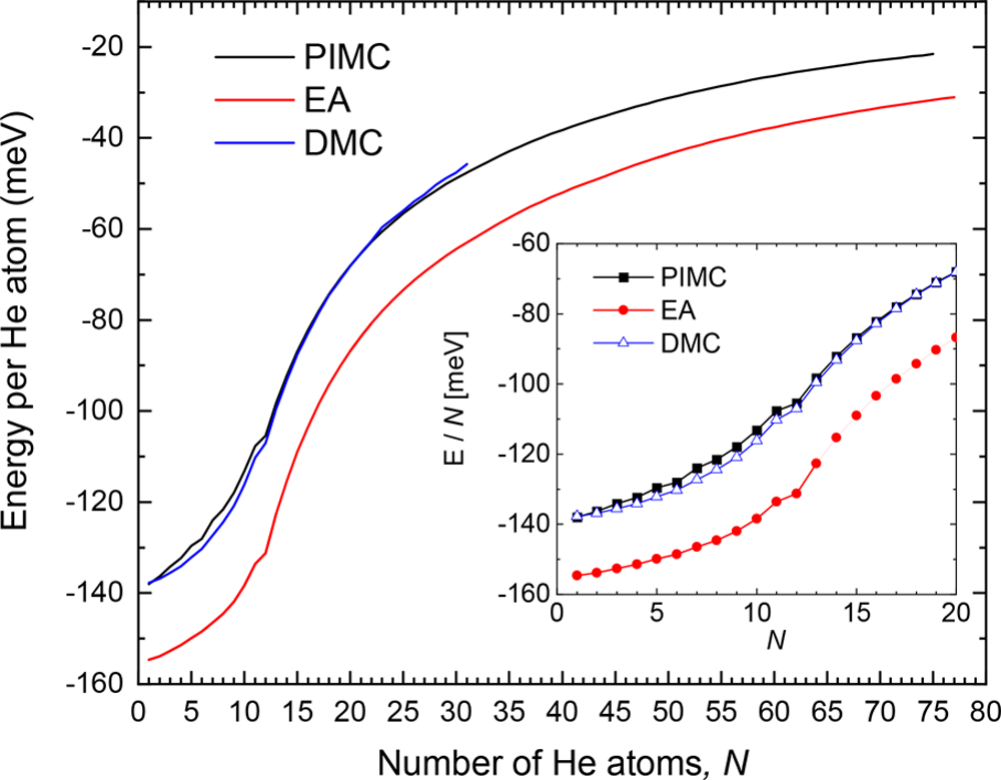
Energy of He_*N*_Ca^2+^ per He atom (in meV) obtained with an evolutionary algorithm (red line), with DMC (blue line), and with PIMC at *T* = 2 K (black line).

**Figure 5:**
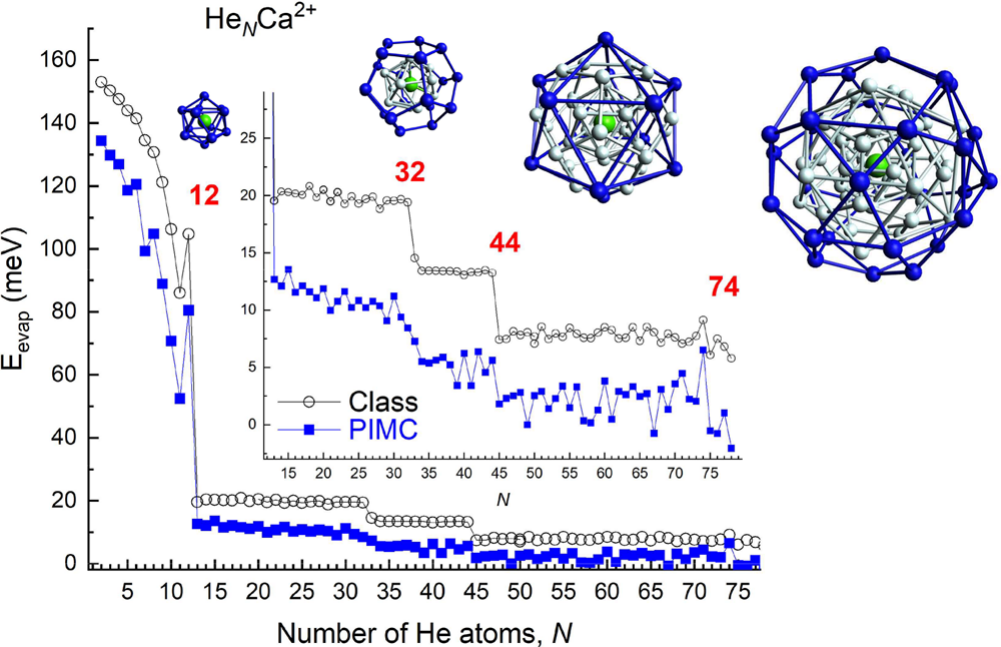
Evaporation energies, defined as *E*_evap_ = *E*_*N*–1_ – *E*_*N*_, in meV, of the different He_*N*_Ca^2+^ droplets as a function of *N*, obtained by means of classical (BH/EA) optimization algorithms (black empty circles) and a PIMC calculation (blue full squares). The region beyond *N* = 13 has been amplified in an inset in order to distinguish the trend followed as a function of *N*. Also, closed shell structures for *N* = 12, 32, 44, and 74, where external He atoms around the Ca^2+^ impurity have been depicted as blue balls (see text for details) have been included.

Moreover, consistent with findings reported in ref (19), the clusters for *N* = 32 and 44 also display closed geometrical structures: on the one hand, the former corresponds to a dodecahedron (shown with blue atoms in the second structure from the left in the inset of Figure 5) containing the icosahedron seen for *N* = 12 as an inner structure, and on the other hand, the latter, He_44_Ca^2+^, is formed in turn with a larger icosahedron (shown in blue atoms in the third structure from the left in the inset of Figure 5) containing the other two structures described, respectively, for *N* = 32 and *N* = 12.

The calculation of the evaporation energies, defined as *E*_evap_ = *E*_*N*–1_ – *E*_*N*_, reveals that such specific cluster sizes are certainly milestones in the growth of He atoms around the Ca^2+^ ion. Figure 5 shows results of calculations performed with both classical and PIMC methods as a function of the number of He atoms. Theoretically estimated *E*_evap_ exhibits sudden drops at the same values of *N* where anomalies are seen in the experimental ion abundances (see Figure 3) suggesting the existence of distinct solvation shells. Thus, regions A and B of Figure 3 match the plateaus observed for the classical evaporation energies in Figure 5 around ∼20 and 13 meV, corresponding to the filling of the second and third shells, respectively. Such a nice agreement between experiment and theory has also been reported in the already mentioned investigation for He_*N*_Ar^+^.^9,19^ However, the present challenge in this case is to provide a similar explanation for the region C of the ion abundances and the peak seen at *N* = 74. And, in fact, Figure 5 reveals that beyond *N* = 44 the evaporation energies remain almost constant around ∼7 meV up to *N* = 74 where another closed geometrical structure can be found.

The compact arrangement responsible of the peak for He_74_Ca^2+^ displays an icosidodecahedron formed by 30 outermost He atoms (in blue in the structure at the top right corner of Figure 5) located above the middle points of the edges separating adjacent atoms of the immediately inner icosahedron found at *N* = 44. The structure of this fourth shell thus differs from the dodecahedron that one would expect at *N* = 64 if the same growth pattern would have remained.

The PIMC calculation, also included in Figure 5, confirms, following a more oscillating trend as a function of *N*, the stability regions seen in the classical evaporation energies and interestingly reproduces the peak at *N* = 74. Insight on this four-shell arrangement is gained from the radial distributions for the He–Ca^2+^ distance calculated by means of the PIMC simulation for the cases of *N* = 74 and 75 He atoms (see Figure S4 in the Supporting Information). These theoretical results thus indicate the formation of this structure consisting of up to four closed geometrical structures around Ca^2+^ in a similar manner as the different layers of a Mozartkugel sweet.

A key feature affecting the formation of helium snowballs is related with the interparticle distance at the minimum of the interaction potential between He and the impurity and its comparison with the corresponding He–He one (2.97 Å).^19^ In this sense, the He–Ca^2+^ interaction displays a minimum in the same range as He–He (see Figure S2 in the Supporting Information), at 2.36 Å, quite similar to the distances for the He–Na^+^ (2.31 Å), He–Ar^+^ (2.57 Å), and He–K^+^ (2.86 Å)^16,47,48^ systems for which magic numbers at *N* = 12, 32, and 44 have been also theoretically predicted. The same sequence of specially stable cluster sizes seen for He_*N*_Au^+^ clusters seems to be also related with the similarities between the *R*_He–He_ and ion-He distances.^49^ Although we cannot rule out that a fourth shell might be observed with the new setup for the above-mentioned systems, what makes He_*N*_Ca^2+^ special is its large He–Ca^2+^ well potential depth, 154.6 meV, in comparison with He–Na^+^ (38.3 meV), He–Ar^+^ (∼34 meV), and He–K^+^ (18.2 meV). This attractive interaction seems to be then responsible of the formation of the four shells of helium atoms around Ca^2+^.

Differences in the interaction between He and a monocation or dication for a given element (the former has a shallower potential well and a larger bond distance due to the extra electron which pushes away the He atom) lead to a different number of He atoms required to close the first shell. Thus, for example, in the case of Pb^+^ and Pb^2+^,^15^ for He_*N*_Pb^+^ ^7^ there is a magic number at *N* = 17 and uncertainties regarding the closing of the second layer of He atoms suggesting its liquid-like behavior.^50^ In turn, for He_*N*_Pb^2+^ an icosahedral structure has been suggested at *N* = 12.^7^ The strength of the He–Ca^+^ interaction (4.6 meV,^51^ see Figure S2 of the Supporting Information), smaller than the presently investigated He–Ca^2+^ case (154.6 meV), is also responsible of differences in the corresponding solvated He ions: As opposed to He_*N*_Ca^2+^, where abrupt steps in the ion abundance correspond to well-defined structures, the He_*N*_Ca^+^ complexes seem to present a gradual closing of the first shell between *N* = 17 and *N* = 25 compatible with a liquid-like nature of the clusters.^51^

HNDs doped with Kr^2+^ could be good candidates to exhibit similar large ordered structures, since magic numbers at *N* = 12 and 32 have been already observed in the spectra.^52^ Moreover, preliminary calculations reveal that the electronic ground state He-Kr^2+^(^3^P_2_) could be described with an interparticle equilibrium distance at the same range as He–Ca^2+^ with an even deeper potential well depth. However, the high ionization energy of Kr (13.99 eV + 24.35 eV) makes it difficult to Penning ionize Kr^+^ to Kr^2+^ by He* with the present experimental setup. Other candidates such as multiply ionized lanthanides leading to compact closed shell cations could be also considered and it would be interesting to investigate the possible existence of a different growth pattern of He atoms to form closed solid-like structures in doped HNDs.

In summary, we report here the observation of up to four of such solvating ordered structures around Ca^2+^, the largest containing 74 He atoms, by means of a powerful experimental technique. The theoretical analysis reveals that the outermost He atoms form an icosidodecahedron.

## Supporting Information Available

Experimental ion abundances for He_*N*_Ca^+^ and He_*N*_Ca^2+^, details of the potential energy surface employed in the calculations, and radial distributions for He_74_Ca^2+^ and He_75_Ca^2+^ (PDF)
Transparent Peer Review report available (PDF)
