# Supplementary material for: Observation of Multiple Ordered Solvation Shells in Doped Helium Droplets: The Case of HeNCa2+
Source: J Phys Chem Lett. 2023 Mar 23;14(13):3126–31. doi: 10.1021/acs.jpclett.3c00224 (PMC10084467; doi:10.1021/acs.jpclett.3c00224)
Supplement: Supplementary file 1 — jz3c00224_si_001.pdf [file jz3c00224_si_001.pdf]

Observation of Multiple Ordered Solvation Shells in Doped Helium Droplets: The Case of He_NCa^{2+} Supporting Information

Eva Zunzunegui-Bru,[†,‖] Elisabeth Gruber,[*,‡] Teresa Lázaro,[†,⊥] Massimiliano Bartolomei,[†] Marta I. Hernández,[†] José Campos-Martínez,[†] Tomás González-Lezana,[*,†] Stefan Bergmeister,[‡] Fabio Zappa,[‡] Paul Scheier,[‡] Ricardo Pérez de Tudela,[¶] Javier Hernández-Rojas,[§] and José Bretón[§]

†*Instituto de Física Fundamental, IFF-CSIC, Serrano 123, 28006 Madrid, Spain*

‡*Universität Innsbruck, Institut für Ionenphysik und Angewandte Physik, Technikerstraße 25, 6020 Innsbruck, Austria*

¶*Wiemelhauser Str 217, 44799 Bochum, Germany*

§*Departamento de Física and IUdEA, Universidad de La Laguna, 38205, La Laguna, Tenerife, Spain*

‖*Current address: Institute of Food, Nutrition and Health, ETH Zürich, Schmelzbergstrasse 9, 8092 Zürich, Switzerland.*

⊥*Current address: Chemical Engineering, Av. Països Catalans 26 Tarragona, Tarragona 43004, Spain*

E-mail: e.gruber@uibk.ac.at; t.gonzalez.lezana@csic.es

Experimental Ion Abundances

Figure S1 shows the experimental ion abundances extracted from the mass spectrum.

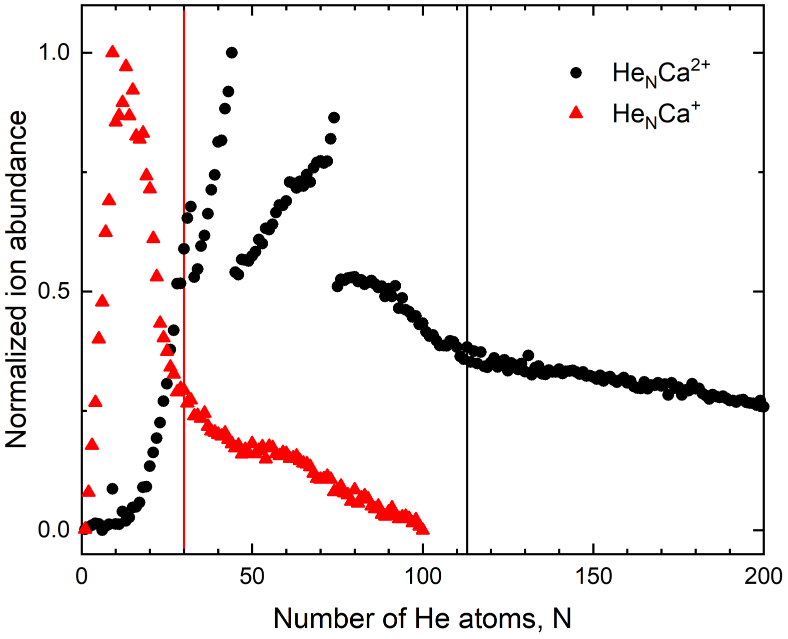

Figure S1: Experimental ion abundances for He_NCa^{2+} (black circles) and He_NCa^{+} (red triangles) clusters as a function of the number of He atoms extracted from the mass spectrum at $P_{\text{evap}} = 0.11$ Pa of Figure 2 of the main manuscript. Ca^{2+} is solvated at average with 113 and Ca^{+} with 30 He atoms, indicated by the vertical lines.

Potential Energy Surface

The theoretical analysis of the doped helium nanodroplets considered in this work requires an accurate description of the interactions among the atoms constituting the droplet. In this study we have followed a similar procedure as in previous investigations of analogous systems.[1] As explained in the main manuscript, the potential energy surface (PES) for the study of the He_NCa^{2+}, is built as the sum of two-body (2B) and three-body (3B) non-covalent interaction contributions. 2B pairwise functions have been considered to describe both the He–He and He–Ca^{2+} interactions: On the one hand, for the first case, we employ the potential by Aziz and Slaman,[2] whereas for the interaction between the He atoms and the dopant, *ab initio* calculated potential energy points have been represented by an analytical form following the improved Lennard-Jones expression:

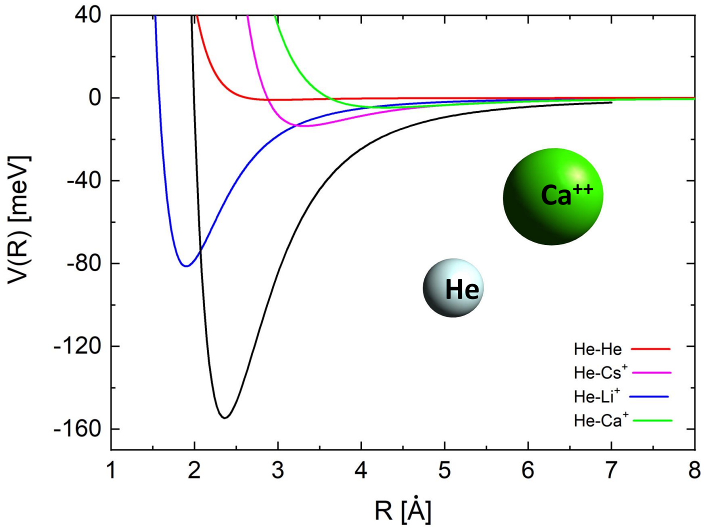

Figure S2: Interaction potential between He and Ca^{2+} (black line) in meV as a function of the interparticle distance He–Ca^{2+} (black) in Å. The figure includes, for comparison, some other potentials between He and different atoms: He (red), Li^{+} (blue), Ca^{+} (green) and Cs^{+} (magenta).

$$V(r) = \epsilon \left[\frac{m}{n(r) - m} \left(\frac{r_m}{r} \right)^{n(r)} - \frac{n(r)}{n(r) - m} \left(\frac{r_m}{r} \right)^{m} \right] \tag{1}$$

with $n(r) = \beta + 4\,(r/r_m)^2$. The values for the corresponding parameters are $m = 4$, $r_m = 2.36$ Å, $\epsilon = 154.615$ meV and $\beta = 5.5$.

The comparison of the actual 2B interactions employed in this study with some other pairwise potentials involving He and some other dopant analyzed before such as Li^{+},[3] Cs^{+},[4] or Ca^{+},[5] shown in Figure S2, reveals that the He–Ca^{2+} interaction is clearly stronger than the rest, with a potential depth of about 154.6 meV (as compared with the values of 81.3, 13.7 and 4.63 meV for He–Li^{+}, He–Cs^{+} and He–Ca^{+}, respectively). In addition to this, many-body effects are found to play a relevant role, as observed in some other complexes involving, for example, Li^{+},[3] or Ba^{2+},[6] ions, with rare gas atoms. Thus, three-body (3B) terms due to the interaction between the dipoles that the dication induces on every pair i, j of the atoms, are added:

$$
\begin{aligned}
V_{ij}^{3B} &= -(q\alpha)^2 \left[\frac{3r_j}{4} g_3(r_i) g_5(r_{ij}) + \frac{3r_i}{4} g_3(r_j) g_5(r_{ij}) \right. \\
&- \frac{1}{4} g_3(r_i) g_3(r_j) g_1(r_{ij}) - \frac{3}{2} g_1(r_i) g_1(r_j) g_5(r_{ij}) \\
&- \left. \frac{1}{2} g_1(r_i) g_3(r_j) g_3(r_{ij}) - \frac{1}{2} g_3(r_i) g_1(r_j) g_3(r_{ij}) \right] \qquad (2)
\end{aligned}
$$

where $\alpha = 1.45\, a_0^3$ is the polarizability of He and $q = 2e$ is the charge of the dication; each r_i represents the distance between the i-th He atom and the Ca^{2+} ion and r_{ij} is the interparticle distance between the i-th and j-th He atoms. In Eq. (2), $g_n(r_i) = f_n(r_i) r_i^{-n}$ where $f_n(r)$ are damping functions defined as:

$$
f_n(r) = 1 - exp(-br) \sum_{k=0}^{n} \frac{[br]^k}{k} \qquad (3)
$$

where $b = 2.91\, a_0^{-1}$ or $2.487 \quad a_0^{-1}$, for r representing the He–Ca^{2+} or He–He distance, respectively.

Additional supermolecular *ab initio* calculations have been performed at the same level as described in the main manuscript in order to obtain total intermolecular interaction energies for the He_2Ca^{2+} and He_3Ca^{2+} clusters. Such interaction energies, obtained by freezing the $HeCa^{2+}$ distance at its equilibrium value and as a function of the He-Ca-He and of the umbrella motion angles for He_2Ca^{2+} and He_3Ca^{2+}, respectively, are reported in Figure S3. We show there a comparison with the analytical representation of the intermolecular interaction for both 2B and 2B+3B models and it can be appreciated that the description of many-body effects by just considering the main 3B term, i.e. that (see Eq. 2) considering the interaction between the dipoles induced by the dication on couples of helium atoms, appears to be a good approximation. As an example, in the region of the He_3Ca^{2+} minimum ($\theta \sim 75°$) a difference between the total *ab initio* energies and the 2B+3B analytical estimations of about 0.5 % is estimated.

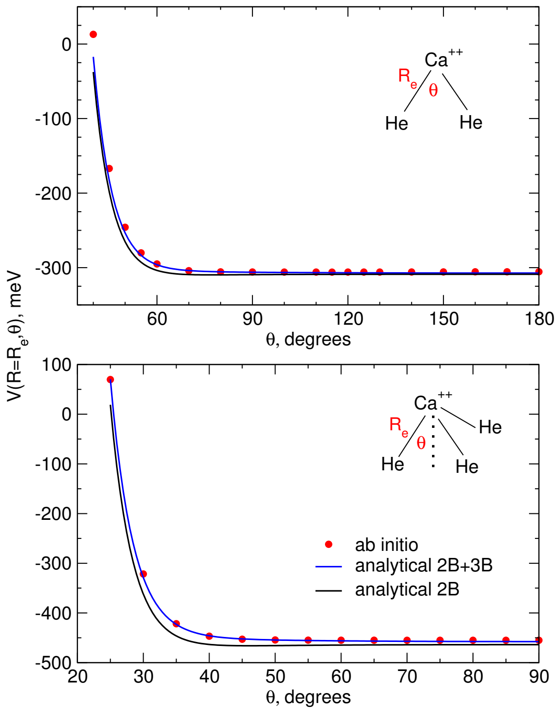

Figure S3: Total intermolecular interaction energies for the He_2Ca^{2+} (upper panel) and He_3Ca^{2+} (lower panel) clusters. *Ab initio* results have been obtained at the CCSD(T)/ d-aug-cc-AV6Z/def2-AQZVPP level of theory (see main manuscript) by freezing the $HeCa^{2+}$ distance at the equilibrium value R_e=2.381 Å and as a function of the θ angle. Analytical results correspond to 2B and 2B+3B representations of the intermolecular interaction.

Radial distributions

Radial distributions for the He–Ca^{2+} distance have been obtained for the $He_{74}Ca^{2+}$ and $He_{75}Ca^{2+}$ cases. In Figure S4 we compare the results obtained from the classical structure employed as the initial configuration for the PIMC calculation and those obtained by means of an average of snapshots stored during the simulation. The comparison between the distributions for the two droplets revealed the existence for the case of $N = 75$ of a small peak around 7.7 Å which corresponds to the extra He atom outside the four closed shells seen for $He_{74}Ca^{2+}$. The quantum distributions obtained by means of the PIMC simulation show

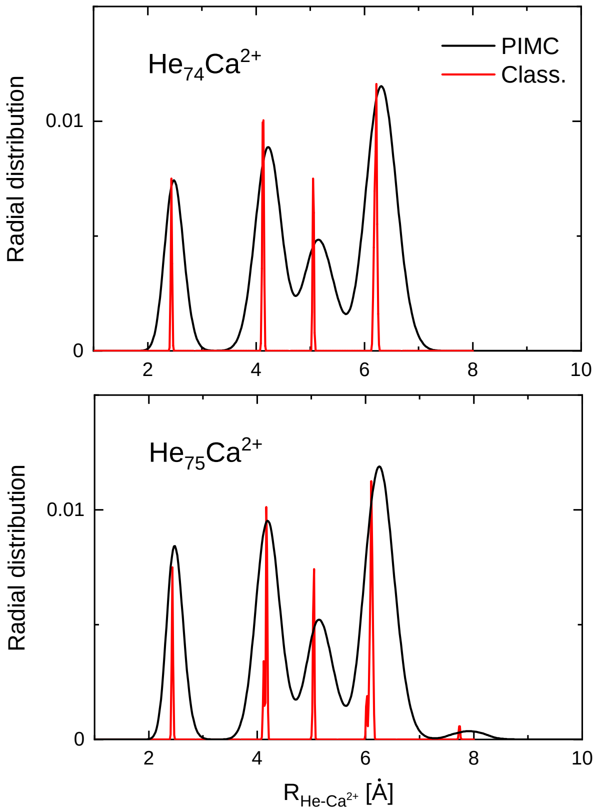

Figure S4: Radial distributions for the He–Ca^{2+} distance (in Å) for the $He_{74}Ca^{2+}$ (upper panel) and $He_{75}Ca^{2+}$ (bottom panel) calculated from the classical configuration (red line) and obtained from the PIMC simulation (black line) as the average from snapshots.

for both sizes, $N = 74$ and 75, certain delocalization of the second, third and fourth shells with respect to the classical structure. Although a complete study of the possible liquid-like behaviour of these shells would require the analysis of parameters such as superfluid fraction, Lindermann index of radius of gyration, present results suffice to show a more delocalized behaviour of the He atoms of the most external shells. The atoms filling the first shell seem to be more confined and thus the corresponding peak seen in the PIMC simulation agrees well with the classical configuration.

References

(1) González-Lezana, T.; Echt, O.; Gatchell, M.; Bartolomei, M.; Campos-Martínez, J.; Scheier, P. Solvation of Ions in Helium. *Int. Rev. Phys. Chem.* **2020**, *39*, 465–516.

(2) Aziz, R. A.; Slaman, M. J. An Examination of Ab Initio Results for the Helium Potential Energy Curve. *J. Chem. Phys.* **1991**, *94*, 8047.

(3) Rastogi, M.; Leidlmair, C.; An der Lan, L.; Ortiz de Zárate, J.; Pérez de Tudela, R.; Bartolomei, M.; Hernández, M. I.; Campos-Martínez, J.; González-Lezana, T.; Hernández-Rojas, J.; Bretón, J.; Scheier, P.; Gatchell, M. Lithium Ions Solvated in Helium. *Phys. Chem. Chem. Phys.* **2018**, *20*, 25569–25576.

(4) Pérez de Tudela, R.; Martini, P.; Goulart, M.; Scheier, P.; Pirani, F.; Hernández-Rojas, J.; Bretón, J.; Ortiz de Zárate, J.; Bartolomei, M.; González-Lezana, T.; Hernández, M. I.; Campos-Martínez, J.; Villarreal, P. A Combined Experimental and Theoretical Investigation of Cs^+ Ions Solvated in He_N Clusters. *J. Chem. Phys.* **2019**, *150*, 154304.

(5) Bartolomei, M.; Martini, P.; Pérez de Tudela, R.; González-Lezana, T.; Hernández, M. I.; Campos-Martínez, J.; Hernández-Rojas, J.; Bretón, J.; Scheier, P. Ca^+ Ions Solvated in Helium Clusters. *Molecules* **2021**, *26*, 3642.

(6) Abdessalem, K.; Habli, H.; Ghalla, H.; Yaghmour, S.; Calvo, F.; Oujia, B. Many-body Effects on the Structures and Stability of $Ba^{2+}Xe_n (n = 1 - 39, 54)$ Clusters. *J. Chem. Phys.* **2014**, *141*.
