# Supplementary material for: Observation of Multiple Ordered Solvation Shells in Doped Helium Droplets: The Case of HeNCa2+
Source: J Phys Chem Lett. 2023 Mar 23;14(13):3126–31. doi: 10.1021/acs.jpclett.3c00224 (PMC10084467; doi:10.1021/acs.jpclett.3c00224)
Supplement: Supplementary file 2 — jz3c00224_si_002.pdf [file jz3c00224_si_002.pdf]

jz-2023-002244.R1

Name: Peer Review Information for "Observation of Multiple Ordered Solvation Shells in Doped Helium Droplets: The Case of $He_NCa^{2+}$"

First Round of Reviewer Comments

Reviewer: 1

Comments to the Author

jz-2023-002244

Observation of Multiple Ordered Solvation Shells in Doped Helium Droplets: The Case of $He_N$ $Ca^{2+}$ by Eva Zunzunegui-Bru et al.

The authors describe experimental work that reveals, for the first time, as many as four solid-like helium solvation shells surrounding an embedded ion. This is by far the largest so-called snowball identified thus far. The experimental data combined with high-level theoretical work reveal that the He atoms in the first three shells are located at the vertices of an icosahedron, dodecahedron, and icosahedron, respectively (as observed before for a few other ions). The solvent atoms in the fourth shell, however, occupy the vertices of an icosidodecahedron.

The experiment is not just a natural extension of previous work; it presents a tour de force. The authors argue that the choice of a dication, $Ca^{2+}$, was instrumental in the formation of a fourth sold-like shell. That choice, however, results in technical challenges. First, the yield of dications tends to be much lower than that of monocations. Second, the presence of dications results in very congested spectra, requiring a very high mass resolution. The authors have met the challenges by designing a novel approach to form doped helium cluster ions with the result that the yield of dications exceeds that of monocations, and by increasing the mass resolution of their instrument by a large factor, to 15 000.

This work presents a significant advance in our understanding of ion solvation in helium droplets. It is suitable, if appropriately revised, for publication in the Journal of Physical Chemistry Letters.

I have no major objections, but the authors may want to consider the following two points. First, on page 11: Snowballs containing three solid-like solvation shells have been reported before ($He_NNa^+$, $He_NAr^+$ etc.). It is certainly possible that a forth shell of icosahedral symmetry in these systems (either at N = 64 or 74) has escaped detection because of low ion yield, insufficient resolution, etc.. Do published mass spectra rule out a forth shell, or do they merely stop below N = 64?

Second, on page 12: If one accepts the (reasonable) assertion that dications enhance the contrast in the evaporation energies, thus leading to more pronounced anomalies in the experimental size distributions, trications may be even better candidates. How about triply charged ions of the rare earths? Would their sizes be about right? Would their ionization energies be low enough to efficiently produce trications by Penning ionization?

There are several smaller issues. Some merely concern language mechanics but others impede understanding.

1. Abstract: change “an improving” to “an improvement”

2. Page 2, 2nd paragraph, “an effective manner to investigate if the formation of shells is the origin of such magic numbers…” – This suggests that there may be alternative explanations for the appearance of such magic numbers? Such as?

3. Page 2, last paragraph: different anomalies => various anomalies

4. Page 2, first line, “the same sequence of magic numbers at N = 12, 32 and 44 have been” => HAS been

5. Further down: prior to pickup the => prior to pickup OF the

6. Following sentence, “enables to tune the specific sizes…” - What is meant by specific size? Probably the size distribution? Rephrase, and insert “us” after “enables”.

7. Further down, “For the case of $Ca^{2+}$ we find… to understand…” - a cumbersome sentence.

8. Further down, and later: containing at average => containing ON average

9. Figure 1, Caption: nucleation sides => nucleation sites

10. Page 4, last line: As in => as described in

11. Fig. 2: The lines tracing the full spectrum disappear when printing in BW. Use thicker lines.

12. Page 6: with ejected charge centers => with these ejected ions (bare charge centers are not ejected!)

13. Two lines below, change “binding energies” to “evaporation energies.” The binding energies (= $-E_N$ ???) change monotonically with N. Hence they trivially differ from those of their neighbors.

Also: The term energy appears in too many different ways: 1. The quantity $E_N$ is never explicitly defined. 2. The ordinate in Fig. 4 is labeled “Energy per He atom”, but in the caption it is called “Interaction energy per He atom.” This is presumably, $E_N$, while the “binding energy” (near the bottom of on page 6) probably refers to $-E_N$.

14. Following paragraph: distinguish He… => distinguish between He….

Also: typically measured => typical

15. Page 7, first line: Fig. 3 displays the ion abundance, not the ion yield (which is plotted in Fig. 2). The former is derived from the latter using suitable software. In the presence of contaminants, the two quantities may have very different size dependences. The authors should the manuscript for every occurrence of the phrase

“yield.”

16. Same paragraph, “Similar ion counts..” is cryptic. Similar ion yields? Certainly not. So, what is similar?

17. Same paragraph, “Different regions can be seen…” Cryptic

18. Page 8: at the low size droplets region => IN the low size droplets region

19. Figure 5: The numbers 32 and 44, which presumably refer to the structures depicted as well as anomalies in the graph, are not well placed.

20. Page 10: “The theoretical estimates for $E_{evap}$ agree with the experimental ion yields” – How can an energy possibly agree with a (unitless) ion yield? What is the expected relation between the size dependences of $E_{evap}$ and the ion abundance? The authors probably refer to the ANOMALIES in the distributions.

21. Further down: “region A and B of Figure 3 are perfectly compatible with the plateaus” – Cryptic. The ion abundance in those regions strongly increases with N, unlike the nearly constant evaporation energies.

22. Further down: “the geometrical structure for N = 74, found just 2 meV above the global minimum” – Cryptic. The global minimum of what? Are you talking about the 74-mer with 2 meV excess energy?

23. Further down: “Otherwise, the next stable closed structures would be at N = 64 and N = 76.” Correct, but difficult to follow for the uninitiated reader.

24. Further down: “the immediately inner icosahedron” – cryptic, rephrase.

25. Further down: consisting in up to four => consisting OF up to four.

26. Page 11, “This attractive interaction must be then responsible” – suggestive, but hardly a proof.

27. Next paragraph, “Differences in the interaction between He and a monocation or dication…” – Insert “For a given element," at the beginning of this sentence.

28. Ref. 14: He4 => $^{4}He$

Reviewer: 2

Comments to the Author

This is a really good manuscript. It absolutely represents the cutting edge in terms of solvated ion studied at low temperatures. The investigation of helium solvation shells around a central ion (an Atkins snowball) is taken to the next level. The observation of four distinct shells being observed for helium-solvated $Ca^{2+}$ is unique. The balance of forces and the particle sizes must be close to optimum to make this possible, and indeed it is noteworthy that $Ca^{+}$ shows a far less extensive shell structure, and indeed evidence for almost liquid solvation ‘shells’. This work is deserving of publication in JPCL for several reasons, including its novelty, the technical achievement, and most importantly because it will prompt future studies, both experimental and theoretical.

I have a number of optional points for the authors to consider and a few minor corrections, and follows:

<u>Optional</u>

How should we view the four shells – as solid or partially liquid-like? Is this revealed in your calculations? If not, care to speculate?

The ‘closure’ of the 4th shell at *N* = 74 is remarkably clear, which begs the question about whether there might be even higher shell closings (5th shell, etc.). Did the authors try this? It’s definitely worth adding a comment either way, as it is quite a tantalising thought.

Although the authors use multiply charged helium droplets in these experiments, there is presumably no particular advantage derived from the multiple charge itself, as the charge transfer to Ca to make the dication can only come from one charge centre in what is presumably a single step. Maybe that’s obvious but I wonder if the authors might want to clarify that?

Corrections

I was confused by the penultimate paragraph on page 10, where the geometrical structure for *N* = 74, is discussed. I cannot see what “global minimum” is being referred to there and I also couldn’t see where “smaller droplets” figures. Equally, the reasoning behind the claims about *N* = 64 and 76 as the next most stable structures is opaque and I cannot see how that relates to the information in Figure 5. A bit more clarification is needed by the authors here.

Reviewer: 3

Comments to the Author

The manuscript by Zunzunegui-Bru reports on a joint theoretical and experimental study on the solvation structure of Ca2+ cations in liquid helium. The experiments use helium droplets in combination with a collision cell to investigate the stability of Ca2+HeN clusters. The experiments reveal the existence several particularly stable structures, most notably one corresponding to 74 helium atoms. The theoretical calculations employing high level ab initio calculations for the interaction potentials are used to find the minimum energies and corresponding structures as a function of cluster size. These calculations are supported by Monte Carlo and Path Integral Monte Carlo quantum mechanical approaches. The theorical results agree with the increased stability of the experimentally observed clusters and provide insight into their geometrical structures. The largest structure, Ca2+He74, appears to consist of four solvation shells, the outer being icosidodecahedro build upon the structures of the smaller stable clusters.

While this is certainly a nice study with valid results, I fail to see the what fundamentally new insight it provides. Both the experimental and theorical work is based upon existing techniques and methods, and the formation of solvation structures involving helium has been extensively studied in the past, both theoretically and experimentally. The fact that in case of Ca2+ up to four solvation layers can be identified in my view is not of broad enough interest to warrant publication in Journal of Physical Chemistry Letters.

Reviewer: 4

Comments to the Author

Referee report on jz-2023-002244

Observation of multiple ordered solvation shells in doped helium
clusters: The case of He_N Ca^2+
by E. Zunzunegui-Bru et al.

This is a combined experimental/computational modeling work reporting a clear evidence for multiple, concentric shells of helium atoms behaving nearly classically, owing to the strong interactions they have with the dication at the center.

The experiment follows a long tradition in one of the most prominent groups in which helium nanodroplets are doped with (sometimes exotic) ions or molecules and studied by accurate mass spectrometry. Here a clever twist consists of starting from multiply charged, large droplets and removing a significant fraction of the size and charge, leading to well controlled doubly charged complexes with a single calcium dication.

Experimental assistance is always needed in these kinds of experiment, provided here by a rather standard approach consisting of an atomistic exploration of the potential energy landscapes by means of classical and quantum methods. The results convincingly show that the magic numbers found at sizes 12, 32 and 44 correspond to the formation of highly symmetric shells that belong to the Ih point group, and they further suggest yet another shell being completed at size 74.

I think those results are original, interesting and in my opinion worthy of publication in JPC as a Letter.

However, I have a few comments that I think should be addressed before the paper can be published in its final form.

(i) In both experiment and modeling the size range studied ends rather shortly after size 74. In the experimental ion yield (fig 3) it seems clear that 74 is indeed special and could correspond to some shell closing. In the calculations (fig 5) it is not so clear, because the

data before and after 74 do exhibit significant fluctuations. I found the discussion of the structure at size 74 not entirely clear, except that it deviates from the global minimum structure (by 2 meV in energy). Is the structure that is being referred to an average configuration produced by the PIMC calculation? Or, conversely, does the PIMC calculation identifies a single structure at this size?

While I agree that the PIMC data suggest a drop in the evaporation energy between 74 and 75, it would have been nice to extend the calculations a bit further, to confirm that this drop is not a local fluctuation.

(ii) I see a couple of potential issues with the semiempirical potential the authors have been developing and using:

-the many-body polarization energy is truncated at order 3, namely the interaction between dipoles induced on two helium atoms. While polarization energy is undoubtedly more significant here due to the dication, it is unclear whether truncating the polarization energy at order 3 (instead of N, or the number of helium atoms), with induced dipoles that are fixed in magnitude, is a realistic approximation. Could the author quantify the error made in neglecting the (exact) self-consistent polarization, and show that this error is smaller than the helium-helium binding energy?

-The polarizability of helium (1.45a_0^3) is said to have been 'multiplied by 2 due to the charge of the dication'. But this puzzles me, as I would expect the polarizability to be intrinsic to the atom, and the charge to influence the polarization energy through the electric fields on the polarizable site. I believe this was only a typo, rather than a genuine ad hoc modification of the polarizability of the (neutral) helium atom.

Author's Response to Peer Review Comments:

**Response to Referee #1**

*The authors describe experimental work that reveals, for the first time, as many as four solid-like helium solvation shells surrounding an embedded ion. This is by far the largest so-called snowball identified thus far. The experimental data combined with high-level theoretical work reveal that the He atoms in the first three shells are located at the vertices of an icosahedron, dodecahedron, and icosahedron, respectively (as observed before for a few other ions). The solvent atoms in the fourth shell, however, occupy the vertices of an icosidodecahedron.*
*The experiment is not just a natural extension of previous work; it presents a tour de force. The authors argue that the choice of a dication, $Ca^{2+}$, was instrumental in the formation of a fourth sold-like shell. That choice, however, results in technical challenges. First, the yield of dications tends to be much lower than that of monocations. Second, the presence of dications results in very congested spectra, requiring a very high mass resolution. The authors have met the challenges by designing a novel approach to form doped helium cluster ions with the result that the yield of dications exceeds that of monocations, and by increasing the mass resolution of their instrument by a large factor, to 15000*.

*This work presents a significant advance in our understanding of ion solvation in helium droplets. It is suitable, if appropriately revised, for publication in the Journal of Physical Chemistry Letters.*

*I have no major objections, but the authors may want to consider the following two points. First, on page 11: Snowballs containing three solid-like solvation shells have been reported before ($He_NNa^+$, $He_NAr^+$ etc.). It is certainly possible that a forth shell of icosahedral symmetry in these systems (either at N = 64 or 74) has escaped detection because of low ion yield, insufficient resolution, etc.. Do published mass spectra rule out a forth shell, or do they merely stop below N = 64?*

Published results for $Ar^+$ in Ref. [9] stop below $N = 60$, but we agree that some of the systems could show larger solvation shells which might have escaped detection in earlier measurements. With the new setup we should be able to detect them in case they exist. However, with the present setup we cannot see larger solvation shells for singly charged Ca cations. Thus, in conclusion, the enhanced binding energy of He to multiply charged impurities is essential too. In fact, the proposed possible candidates to exhibit this multiple-shell structure have an interaction with helium with a sufficiently large potential well depth. A comment on this direction has been introduced in page 11.

*Second, on page 12: If one accepts the (reasonable) assertion that dications enhance the contrast in the evaporation energies, thus leading to more pronounced anomalies in the experimental size distributions, trications may be even better candidates. How about triply charged ions of the rare earths? Would their sizes be about right? Would their ionization energies be low enough to efficiently produce trications by Penning ionization?*

Indeed, for future investigations we plan to move towards triply charged ions. Our candidate of choice are lanthanides as their ionization energies are low enough to produce trications via sequential Penning ionization in helium nanodroplets. This has been mentioned now at the end of the letter.

*There are several smaller issues. Some merely concern language mechanics but others impede understanding.*

*1. Abstract: change "an improving" to "an improvement"*

This has been removed after shortening the abstract.

*2. Page 2, 2nd paragraph, "an effective manner to investigate if the formation of shells is the origin of such magic numbers…" – This suggests that there may be alternative explanations for the appearance of such magic numbers? Such as?*

There are cases in which maximum features in experimental ion yields have been found to correspond simply to especially stable configurations with respect to close neighbor sizes of the He atoms solvating the dopant. See for instance the case of $He_NLi^+$ in Rastogi *et al. Phys. Chem. Chem. Phys.* **2018**, *20*, 25569.

*3. Page 2, last paragraph: different anomalies => various anomalies*

This has been changed

*4. Page 2, first line, "the same sequence of magic numbers at N = 12, 32 and 44 have been" => HAS been*

This has been changed

*5. Further down: prior to pickup the => prior to pickup OF the*

This has been corrected

*6. Following sentence, "enables to tune the specific sizes…" - What is meant by specific size? Probably the size distribution? Rephrase, and insert "us" after "enables".*

This has been corrected

*7. Further down, "For the case of $Ca^{2+}$ we find… to understand…" - a cumbersome sentence.*

This sentence has been rephrased for the sake of clearity

*8. Further down, and later: containing at average => containing ON average*

This has been corrected

*9. Figure 1, Caption: nucleation sides => nucleation sites*

This has been corrected

*10. Page 4, last line: As in => as described in*

This has been corrected

*11. Fig. 2: The lines tracing the full spectrum disappear when printing in BW. Use thicker lines.*

The figure has been changed

*12. Page 6: with ejected charge centers => with these ejected ions (bare charge centers are not ejected!)*

This has been corrected

*13. Two lines below, change "binding energies" to "evaporation energies." The binding energies (= -$E_N$ ???) change monotonically with N. Hence, they trivially differ from those of their neighbors. Also:*

*The term energy appears in too many different ways: 1. The quantity $E_N$ is never explicitly defined. 2. The ordinate in Fig. 4 is labeled "Energy per He atom", but in the caption it is called "Interaction energy per He atom." This is presumably, $E_N$, while the "binding energy" (near the bottom of on page 6) probably refers to $-E_N$.*

The referee is right that the identification of the different energies is not clear in the text. We have now introduced $E_N$, in pg. 7 when energies per atom are defined. The caption for Figure 4 has also been corrected.

*14. Following paragraph: distinguish He… => distinguish between He…. Also: typically measured => typical*

This has been corrected

*15. Page 7, first line: Fig. 3 displays the ion abundance, not the ion yield (which is plotted in Fig. 2). The former is derived from the latter using suitable software. In the presence of contaminants, the two quantities may have very different size dependences. The authors should the manuscript for every occurrence of the phrase "yield."*

"*Yield*" has been replaced by "*Abundance*" in Figure 3, in the y-labels of both the main figure and the inset and in the corresponding caption. We have checked it along the text similar change has been done.

*16. Same paragraph, "Similar ion counts.." is cryptic. Similar ion yields? Certainly not. So, what is similar?*

The sentence has been rephrased to make clear that ion abundances obtained with selection of the size distribution were also reported recently in Reference (33)

*17. Same paragraph, "Different regions can be seen…" Cryptic*

We have also rephrased the sentence to point out that the observed peaks separate sizes in four different ranges.

*18. Page 8: at the low size droplets region => IN the low size droplets region*

This has been corrected.

*19. Figure 5: The numbers 32 and 44, which presumably refer to the structures depicted as well as anomalies in the graph, are not well placed.*

Labels with numbers 32 and 44 have been moved in Figure 5

*20. Page 10: "The theoretical estimates for $E_{evap}$ agree with the experimental ion yields" – How can an energy possibly agree with a (unitless) ion yield? What is the expected relation between the size dependences of $E_{evap}$ and the ion abundance? The authors probably refer to the ANOMALIES in the distributions.*

The referee is right, the sentence is misleading. We have changed it to say that anomalies in the evaporation energy as a function of *N* the number of He atoms match the magic numbers observed in the ion abundances.

*21. Further down: "region A and B of Figure 3 are perfectly compatible with the plateaus" – Cryptic. The ion abundance in those regions strongly increases with N, unlike the nearly constant evaporation energies.*

The referee is right that the text was a bit obscure. We have rephrased that part indicating that both experimental ion abundances and theoretical evaporation energies show anomalies at the same cluster sizes that we interpret as the filling of the second and third solvation shells.

*22. Further down: "the geometrical structure for N = 74, found just 2 meV above the global minimum" –Cryptic. The global minimum of what? Are you talking about the 74-mer with 2 meV excess energy?*

What we want to say is that the geometrical structure shown for *N*=74 does not correspond to the global minimum obtained with a classical optimization algorithm. The difference between the classical energies of such a minimum and that of the structure included in Figure 5 is nevertheless only 2 meV. The sentence has been removed from the text in order to avoid confusion. -

*23. Further down: "Otherwise, the next stable closed structures would be at N = 64 and N = 76." Correct, but difficult to follow for the uninitiated reader.*

We have tried to clarify this point in the new version.

*24. Further down: "the immediately inner icosahedron" – cryptic, rephrase.*

We have indicated that such inner icosahedron corresponds to the structure seen at *N* = 44.

*25. Further down: consisting in up to four => consisting OF up to four.*

This has been now corrected

*26. Page 11, "This attractive interaction must be then responsible" – suggestive, but hardly a proof.*

We have softened the statement by replacing "***must***" by "***seems to***"

*27. Next paragraph, "Differences in the interaction between He and a monocation or dication…" – Insert "For a given element," at the beginning of this sentence.*

This has been inserted

*28. Ref. 14: He4 => $_4$He*

This has been corrected.

**Response to Referee #2**

*This is a really good manuscript. It absolutely represents the cutting edge in terms of solvated ion studied at low temperatures. The investigation of helium solvation shells around a central ion (an Atkins snowball) is taken to the next level. The observation of four distinct shells being observed for helium-solvated Ca2+ is unique. The balance of forces and the particle sizes must be close to optimum to make this possible, and indeed it is noteworthy that Ca+ shows a far less extensive shell structure, and indeed evidence for almost liquid solvation 'shells'. This work is deserving of publication in JPCL for several reasons, including its novelty, the technical achievement, and most importantly because it will prompt future studies, both experimental and theoretical.*

*I have a number of optional points for the authors to consider and a few minor corrections, and follows:*

*Optional*

*How should we view the four shells – as solid or partially liquid-like? Is this revealed in your calculations? If not, care to speculate?*

Radial distributions for the He—$Ca^{2+}$ distance have been calculated for the case of $N$ = 74 and 75 He atoms by means of an average over snapshots taken during the PIMC simulation. Results have been included in Figure S4 in the Supplementary Information. Although a proper analysis of the possible liquid-like behavior of these solvating shells would require the study of proper parameters (such as superfluid fraction, Lindemann index, radius of gyration…), the comparison of the above mentioned radial distributions with the corresponding classical structures reveals interesting features regarding the delocalization of the He atoms on each shell. The shape of the first shell and its comparison with the classical counterpart suggests a solid-like behavior. In turn, the distributions corresponding to the second, third and fourth shells are rather wide but still remain in a fairly good qualitative agreement with the classical estimates. This can be interpreted as an intermediate behavior between solid and liquid-like. According to this, we have decided to refer to the shells as "well-ordered" instead of just "solid-like" along the text.

*The 'closure' of the 4th shell at N = 74 is remarkably clear, which begs the question about whether there might be even higher shell closings (5th shell, etc.). Did the authors try this? It's definitely worth adding a comment either way, as it is quite a tantalising thought.*

We think that for $Ca^{2+}$ there are no more solvating shells beyond the last one we have observed here. The reason to believe this can be found in the actual values of the evaporation energy predicted by the PIMC calculation beyond this fourth shell become negligible and eventually even smaller than the corresponding evaporation energies for pure Helium droplets.

*Although the authors use multiply charged helium droplets in these experiments, there is presumably no particular advantage derived from the multiple charge itself, as the charge transfer to Ca to make the dication can only come from one charge centre in what is presumably a single step. Maybe that's obvious but I wonder if the authors might want to clarify that?*

Using multiply charged droplets increases the number of $He_NCa^+$ ions as upon mild doping we can form under the present conditions typically 5 of these complexes per droplet besides a few

bare He cluster ions, some He solvated Ca dimer and trimer cations. The referee is completely right that in the case of Ca we do not need a second ionizing agent to form a dication since the sum of the first and second ionization energy of Ca is only 17.98 eV. Thus, double ionization of Ca is energetically possible by both $He_N^+$ and He*. In fact, if a second Ca atom attaches to a $Ca^{2+}$, we expect Coulomb explosion into two singly charged Ca ions. Additional pickup of Ca will then lead to the formation of singly charged Ca cluster ions inside the He droplets.

*Corrections*

*I was confused by the penultimate paragraph on page 10, where the geometrical structure for N =74, is discussed. I cannot see what "global minimum" is being referred to there and I also couldn't see where "smaller droplets" figures. Equally, the reasoning behind the claims about N = 64 and 76 as the next most stable structures is opaque and I cannot see how that relates to the information in Figure 5. A bit more clarification is needed by the authors here*.

The referee is right about this. Referee #1 also points out that this part is particularly opaque so we have decided to remove the reference to the global minimum and to rephrase the discussion about possible stable structures beyond $N$ = 44.

**Response to Referee #3**

*The manuscript by Zunzunegui-Bru reports on a joint theoretical and experimental study on the solvation structure of Ca2+ cations in liquid helium. The experiments use helium droplets in combination with a collision cell to investigate the stability of Ca2+HeN clusters. The experiments reveal the existence several particularly stable structures, most notably one corresponding to 74 helium atoms. The theoretical calculations employing high level ab initio calculations for the interaction potentials are used to find the minimum energies and corresponding structures as a function of cluster size. These calculations are supported by Monte Carlo and Path Integral Monte Carlo quantum mechanical approaches. The theorical results agree with the increased stability of the experimentally observed clusters and provide insight into their geometrical structures. The largest structure, Ca2+He74, appears to consist of four solvation shells, the outer being icosidodecahedro build upon the structures of the smaller stable clusters.*

*While this is certainly a nice study with valid results, I fail to see the what fundamentally new insight it provides. Both the experimental and theorical work is based upon existing techniques and methods, and the formation of solvation structures involving helium has been extensively studied in the past, both theoretically and experimentally. The fact that in case of Ca2+ up to four solvation layers can be identified in my view is not of broad enough interest to warrant publication in Journal of Physical Chemistry Letters.*

Contrary to the reviewer's conclusion, we do believe that the discovery of four well-ordered shells of helium around an ion is a remarkable and surprising feature, taking into account the very weak nature of helium as a solvent. In this way, the finding provides a new perspective on the role of helium as a solvent and opens the question about how many other systems might present multiple solvation shells, not observed yet.

Present results have been achieved thanks to a new design and technical improvements of the Innsbruck experimental setup, where a) the use of multiple charged helium nanodroplets allows an increase of the signal; b) specific size distributions of the droplet can be efficiently tuned by modifying the pressure in the evaporation chamber and c) the very high mass resolution (15000). These achievements, together with the guidelines about the most favourable interactions provided by theory, are prompting much more comprehensive studies of the solvation of many different ions (single, double and even triply charged).

**Response to Referee #4**

*This is a combined experimental/computational modeling work reporting a clear evidence for multiple, concentric shells of helium atoms behaving nearly classically, owing to the strong interactions they have with the dication at the center.*

*The experiment follows a long tradition in one of the most prominent groups in which helium nanodroplets are doped with (sometimes exotic) ions or molecules and studied by accurate mass spectrometry. Here a clever twist consists of starting from multiply charged, large droplets and removing a significant fraction of the size and charge, leading to well controlled doubly charged complexes with a single calcium dication.*

*Experimental assistance is always needed in these kinds of experiment, provided here by a rather standard approach consisting of an atomistic exploration of the potential energy landscapes by means of classical and quantum methods. The results convincingly show that the magic numbers found at sizes 12, 32 and 44 correspond to the formation of highly symmetric shells that belong to the Ih point group, and they further suggest yet another shell being completed at size 74.*

*I think those results are original, interesting and in my opinion worthy of publication in JPC as a Letter.*

*However, I have a few comments that I think should be addressed before the paper can be published in its final form.*

*(i) In both experiment and modeling the size range studied ends rather shortly after size 74. In the experimental ion yield (fig 3) it seems clear that 74 is indeed special and could correspond to some shell closing. In the calculations (fig 5) it is not so clear, because the data before and after 74 do exhibit significant fluctuations. I found the discussion of the structure at size 74 not entirely clear, except that it deviates from the global minimum structure (by 2 meV in energy). Is the structure that is being referred to an average configuration produced by the PIMC calculation? Or, conversely, does the PIMC calculation identifies a single structure at this size? While I agree that the PIMC data suggest a drop in the evaporation energy between 74 and 75, it would have been nice to extend the calculations a bit further, to confirm that this drop is not a local fluctuation.*

The structure shown in the inset for Figure 5 does not correspond to a PIMC result. It is a classical configuration which is employed as initial structure for the PIMC simulation. Figure S4 included now in the Supplementary Information shows radial distributions for the He—$Ca^{2+}$ distance for the $He_{74}Ca^{2+}$ and $He_{75}Ca^{2+}$, obtained from the PIMC simulation in comparison with the corresponding classical distributions. The onset of a maximum peak around 7.7 Å in the case of 75 He atoms supports the idea of an external He atom outside the four closed shells seen for the 74 droplet. In our opinion, this constitutes a further confirmation of the closure of the fourth shell at $N$ = 74.

The referee is right about the convenience of extending the PIMC simulations to larger sizes. However, for those clusters $N$ larger than 74 we have analyzed, the values found for the evaporation energies are: (i) of the same order of the intrinsic error of the calculation; (ii) small and (iii) clearly smaller than those seen for the fourth shell.

*(ii) I see a couple of potential issues with the semiempirical potential the authors have been developing and using:*

*-the many-body polarization energy is truncated at order 3, namely the interaction between dipoles induced on two helium atoms. While polarization energy is undoubtedly more significant here due to the dication, it is unclear whether truncating the polarization energy at order 3 (instead of N, or the number of helium atoms), with induced dipoles that are fixed in magnitude, is a realistic approximation. Could the author quantify the error made in neglecting the (exact) self-consistent polarization, and show that this error is smaller than the helium-helium binding energy?*

Additional supermolecular *ab initio* calculations have been performed at the same level as described in the main manuscript in order to obtain total intermolecular interaction energies for the $He_2Ca^{2+}$ and $He_3Ca^{2+}$ clusters. Such interaction energies, obtained by freezing the $HeCa^{2+}$ distance at its equilibrium value and as a function of the He-Ca-He and of the umbrella motion angles for $He_2Ca^{2+}$ and $He_3Ca^{2+}$, respectively, are reported in Figure S3 in the Supplementary Information.

We show there a comparison with the analytical representation of the intermolecular interaction for both two-body (2B) and 2B+3B models and it can be appreciated that the description of many-body effects by just considering the main 3B term, i.e. that considering the interaction between the dipoles induced by the dication on couples of helium atoms, appears to be a good approximation. As an example, in the region of the $He_3Ca^{2+}$ minimum we have estimated a discrepancy between the total ab initio energies and the 2B + 3B analytical estimations of about 0.5 %.

*-The polarizability of helium (1.45a_0^3) is said to have been 'multiplied by 2 due to the charge of the dication'. But this puzzles me, as I would expect the polarizability to be intrinsic to the atom, and the charge to influence the polarization energy through the electric fields on the polarizable site. I believe this was only a typo, rather than a genuine ad hoc modification of the polarizability of the (neutral) helium atom.*

We thank the reviewer for reporting this point and in the revised version of the Supporting Information we have rephrased the corresponding text and corrected the Eq. 2 in order to better formulate the used 3B term.
